# The “Facebook-self”: characteristics and psychological predictors of false self-presentation on Facebook

**DOI:** 10.3389/fpsyg.2015.00099

**Published:** 2015-02-17

**Authors:** Oren Gil-Or, Yossi Levi-Belz, Ofir Turel

**Affiliations:** ^1^College of Management Academic StudiesRishon LeZion, Israel; ^2^The Academic College of Tel Aviv Yafo, Tel Aviv YafoIsrael; ^3^Department of Behavioral Sciences, Ruppin Academic CenterEmek Hefer, Israel; ^4^Information Systems and Decision Sciences, Mihaylo College of Business and Economics, California State UniversityFullerton, CA, USA; ^5^Psychology, Brain and Creativity Institute, University of Southern California, Los Angeles, CAUSA

**Keywords:** social networking sites, Facebook, false self, attachment theory, self-esteem, authenticity

## Abstract

In this study we present and empirically examine a new phenomenon related to social networking sites, such as Facebook, the “false Facebook-self.” Arguably false self-presentation on Facebook is a growing phenomenon, and in extreme cases; i.e., when ones Facebook image substantially deviates from their true image, it may serve as a gateway behavior to more problematic behaviors which may lead to psychological problems and even pathologies. In this study we show that certain users are more vulnerable to such false self-presentation than others. The study involved 258 Facebook users. Applying ANOVA and SEM analyses we show that users with low self-esteem and low trait authenticity are more likely than others to present a Facebook-self which deviates from their true selves. These social-interaction-related traits are influenced by one’s upbringing and the anxious and avoidant attachment styles he or she has developed. Several cases (7.5%) with large gaps between the true and false Facebook-self were detected, which implies that future research should consider the adverse consequences and treatments of high levels of false Facebook-self.

## INTRODUCTION

The use of Facebook has been increasing in the last decade, with tens of millions of new users joining it on a quarterly basis worldwide (Internet World Stats, 2012). Each of these users spends, on average, around 14 min every day on Facebook, which represents 16.6% of the overall time spent online (Comscore1, 2011). This increased use, however, could be a double-edged sword as social networking sites provide their members with utilitarian, social and self-enhancing benefits, but at the same time, can produce negative well-being, work-life balance, and work performance consequences (Turel et al., 2011a,b, 2014; Turel and Serenko, 2012; Turel, 2015). Primarily, Facebook provides its members with the opportunity to interact with many different people, called “Facebook friends.” Through these interactions users, especially those with low self-esteem and low life satisfaction can improve their psychological well-being, (Ellison et al., 2007). However, these rewarding interactions can lead to excessive use of such sites, up to the level of addiction (Turel and Serenko, 2012; Turel et al., 2014), especially for individuals with low self-esteem, low self-efficacy and other “problematic” characteristics (Brand et al., 2014), and those with strong need for escapism (Xu et al., 2012).

In this study we argue and demonstrate that the use of Facebook can also be associated with other psychological problems, beyond addiction. We specifically suggest that the use of Facebook, and perhaps other social media sites, can promote false presentation of the self, which we presume to be unhealthy based on false-self theories (Rogers, 1959; Winnicott, 1960), because it can ultimately lead to reduced wellbeing and promote various psychological pathologies. Unlike other online destinations, such as portals, news websites and even blogs, where one can comment either anonymously or under a fake name, social networking sites require their users to expose themselves, not just in terms of names, but also in terms of friends, activities, images, feelings, and preferences. As a result, some users decide, consciously or unconsciously, to present an identity or a self that deviates from their true-self (if such a thing really exists). Presumably, and assuming rationality, these users self-enhance their true identity to match a desired one, but would unlikely play down their true identity.

False self-presentation, to a certain degree, is not a phenomenon that was invented in the digital age. People often present themselves in a manner which is inconsistent with who they really are and with their set of real beliefs and values. The gap between the real self and the ideal self is called incongruity (Rogers, 1959); and it could lead to the creation of “false self” (Winnicott, 1960) which is a more defensive, protective self that hides one’s “true self.” Both Winnicott and Rogers suggested that low degrees of falseness are natural and perhaps not harmful. However, high degrees of falseness, or in other words, wide and persistent gaps between the true/real self and the false/ideal self, can result in precarious functioning and psychological vulnerability which ultimately can lead to psychological pathologies. We hence believe that such false self-representations on Facebook can lead to similar negative consequences and may merit increased awareness, and in some extreme cases – when the gaps between the true and false self are large and the person adopts his or her own false identity, even warrant counseling and treatment.

As such, we extend the idea of false-self and its consequent psychological vulnerabilities to a new domain, namely Facebook, which provides an efficient vehicle for developing and presenting a false-self. We call this false presentation the “Facebook-self.” The “Facebook-self” is usually a more socially acceptable and popular self, and as such, can be very different from the user’s real or “true self.” The circumstances that lead to the creation of a false “Facebook-self” may differ from one user or situation to another. For instance, there is reason to predict that the difference between the “true-self” and the “Facebook-self” might be bigger when an individual is not satisfied with his or her real life or have low self-esteem and as a result creates an alternative environment to compensate for his or her real-life deficiencies. This is a natural compensation-defensive mechanism which shelters individuals from internal and external threats (e.g., social pressures and expectations) to their true-self. For instance, a recent survey showed that nearly two-thirds of mothers who use social networking sites felt pressure to create a social presence that depicted the perfect life, even though clearly life with newborns is not always perfect, i.e., they create “false selves” on such sites (emarketer, 2014). The creation of a false “Facebook-self” can also be a result of various individual differences in attachment style and authenticity. We consequently develop a theory which ties these concepts and explains the creation of the Facebook-self.

Ultimately, this study focuses on the “Facebook-self” and its predictors, because it is presumed that presenting a false image on a social networking site is easier and less risky that in real life; and consequently it may be prevalent in such environments. Moreover, it may influence the well-being of many users, since a growing percentage of their social lives shifts to online environments, such as Facebook. Hence, some users, especially with vulnerable personalities may create online identities which are much more rewarding and appealing than their true identities. These growing reward may have the potential to increase user risks of engaging in precarious behaviors and even developing addiction to the use of such sites (Turel et al., 2014). As such, we see the false Facebook-self as a possible gateway phenomenon to more problematic behaviors which may lead to psychological problems and pathologies. We hence posit that the phenomenon of false Facebook-self merits research, and might be relevant for treatment in extreme cases.

### THEORY

#### False identity and the Facebook-self

Winnicott’s (1960) theory posits that one’s ego can split to “true self” and “false self.” These terms describe two types of experiences: one is more spontaneous, authentic and real and the other is more defensive and protective, trying to hide the “true self.” Similarly, Rogers’ (1959) theory defined the real self as the underlying organismic self. The ideal self often deviates from the real-self, and this can be a result of the lack of positive regard during childhood. It represents who one would like to be, as a result of the feedback he or she received during their developmental period. The gap between the real self and the ideal self is called incongruity. Both theories also suggest that large gaps or incongruence between the true and protective self can lead to various psychological problems. Behaving according to one’s “true self,” as well as having clear and explicit identity, tends to have positive consequences. This idea extends to online environments. People who present their “true selves” and are authentic on online media, tend to create honest, healthier and longer relationships with their online friends. These relationships, in many cases, are also translated into the oﬄine world (McKenna et al., 2002).

One should note that there are also short-terms advantages to false presentations; and hence presenting a false self can be rational and perceived as a self-advantageous behavior. In a process called “identity play” a person can explore and adopt different identities that are different from his or her own identity (Turkle, 1995). This behavior can be considered positive if it is done from time to time and if it is flexible, meaning the person can freely change his or her behavior in different states. In addition, one’s “false self” protects a person from showing others (including him- or her-self) who he or she really is or how he or she really feels, which in many cases can be a major benefit explaining the common use of “false self” and specifically false Facebook-self in our society. However, these advantages are realized primarily when the false-self is not persistent and is not too far from the true self. Hence, when these conditions are not met, a person may suffer from the negative consequences of his or her falseness.

How can a false-self be created on Facebook? Users on Facebook expose information about their identity in various ways, from their demographic profile information and education/professional experience to photographs, clips and written text. In contrast to anonymous social networks and sites (such as: blogs, forums, and others), it is uncommon for Facebook users to present false surface information (i.e., they mostly present true name, activities, and social demographic information). However, their deep identities are often presented in an implicit way using cues and signals embedded in their posts and images. For example, people may selectively post images of them being happy dining at a fancy restaurant with good looking friends, in an implicit attempt to enhance their image in the eyes of others in their social network; even though their true selves may be depressed and introvert, and the rest of their week was pretty dull. Consequently, users keep their identity much more implicit and create signals that present their identity in a positive way (Zhao et al., 2008). Hence, the Facebook-self in this research is a collection of signals given by a Facebook user to his or her Facebook community. These signals include all information, from the profile information (academic and professional background, hobbies, and others), followed by the uploaded content (photos, songs, and others) and ending with the published communications one posts on his or her “wall” or on other people’s “walls” (messages, referred links, and other pieces of information). In this sense, one-to-one messages sent via Facebook are usually not a part of the public “Facebook-self.” Please note that the “Facebook-self” following Winnicott’s theory, might in some cases represent a “true self.” However, in many cases it is likely to manifest a “false self” to a certain degree, which can diverge from one’s “true self.” This falseness is not necessarily intentional or even conscious, as it can represent one’s non-Facebook “False self.”

Personality characteristics that contribute to the presentation of a false-self, regardless of Facebook, are low self-esteem and unawareness of the true self (Harter et al., 1996), which may be manifested through low general authenticity. We therefore focus on these variables in this study, and apply them to the case of the Facebook-self.

Why should we care about false Facebook-self? There are several negative outcomes which may stem from a false Facebook-self. Specifically, it might negatively affect one’s well-being, especially if done consistently and inflexibly, since authenticity is correlated with both subjective well-being and psychological well-being (Wood et al., 2008). We also assumed that keeping a false “Facebook-self” increases the reward one gets out of Facebook, as it provides a non-realistic environment one can act in, which may serve as a fertile ground for the development of addiction to the use of Facebook (Turel et al., 2014). Hence, therapists may want to deal with extreme cases of Facebook-self in order to prevent such issues. The point at which falseness on Facebook requires treatment is a topic for a different study, and requires further research.

#### Psychological predictors of false Facebook-self

Facebook is the largest social networking site on the Internet, with more than 1.3 billion active users, 829 million daily active users and ∼15% annual growth (Facebook, 2014). People use Facebook for developing and maintaining social ties; and such uses can improve the well-being of individuals by reducing their feelings of loneliness and depression (Kraut et al., 1998). Other possible benefits of using such sites include increases in self-esteem and perceived social support (Ellison et al., 2007; Bessière et al., 2008; Gonzales and Hancock, 2011), as well as enjoyment, satisfaction (Turel and Serenko, 2012; Turel, 2015) and positive affect (Bareket-Bojmel and Shahar, 2011).

Given these social and self-enhancing benefits, the use of such websites may be especially appealing for people with low self-esteem, who can use such sites to self-disclose in a “protected” environment (Reis and Shaver, 1988). Unfortunately, even though such sites look like “safe” environments, recent research shows that interactions with and on such sites can lead to unwarranted consequences. For example, retaliation against people with different opinions may be common on such sites (Forest and Wood, 2012). This unsupportive interaction causes social networking site users with depressive symptoms to have negative interactions and negative affect (Feinstein et al., 2012). This concern regarding social-and affective-safety may be one predictor that promotes social compliance and the development of false Facebook-self.

Furthermore, lonely people that have low social skills tend to develop strong compulsive Internet use behaviors, and as a result have negative life outcomes instead of relieving their original problems (Kim et al., 2009). Similarly, individuals with materialistic values who believe that online purchases will enhance their emotions and identity, develop compulsive shopping tendencies (Dittmar et al., 2007); and others may feel guilty regarding the time they spend on Facebook and the way they manage relationships on Facebook (Turel, 2015). As such, Facebook usage has a range of effects on the self, much beyond the Facebook boundaries. It affects the feelings, attitudes and behaviors of its users in the oﬄine world, outside of Facebook.

As discussed above, Facebook can have positive impacts on certain users and negative effects on others, and it is also possible that it has positive and negative effects on the same users (Tarafdar et al., 2013; D’Arcy et al., 2014). One of the potential causes to this variation in the positive and the negative effects of social networking sites is the wide spectrum of Facebook users who differ in their characteristics, personalities, intensity and forms of Facebook usage, benefits they get from the social networking sites, and attitudes toward Facebook. Analysis of this wide range of personalities, behaviors, attitudes and emotions can shed some light on the various effects Facebook has on its users. Focusing on the characteristics that put certain users at risk might point to possible prevention strategies, and this is the avenue of research we pursue in this study.

The negative effects of Facebook usage were mainly found in parameters such as reduced oﬄine social life participation, withdrawal from academic studies challenges in relationships (Kuss and Griffiths, 2011), and sense of time wasting and guilt feelings (Turel, 2015). It was also found that some Facebook users prefer their social interaction to be online (versus oﬄine). These users usually engage in social networking site use as a means to regulate their mood changes. When they have deficient self-regulation abilities, they may engage in excessive and problematic Facebook use (Lee et al., 2012), which is sometimes classified as an addiction to the use of such sites (Turel and Serenko, 2012; Brand et al., 2014). From a brain activation standpoint, problematic use of Facebook is typically driven by hyper-sensitized and hyperactive amygdala-striatal system (Turel et al., 2014).

In addition to the abovementioned variables, it is possible that the social aspects of Facebook use are associated with its users’ attachment style (Bowlby, 1969), albeit these effects are likely to be indirect and mediated through traits which are developed post-childhood. Attachment theory predicts that early childhood relationships of the infant with his or her main caregiver influence his or her long-term relationships with others. Hence, its relevancy to Facebook use, which is largely about relationship development and maintenance. Attachment theory points to four attachment patterns: secure, avoidant, ambivalent/resistant and disorganized. These styles can influence Facebook use in various ways, and particularly non-secure attachment styles (anxious and avoidant) can be associated with the ways people manage relationships and interact on Facebook: (1) anxious attachment style is positively related to relationships within Facebook and (2) avoidant attachment style is negatively related to these relationships, with expression of jealousy and surveillance on Facebook (Marshall et al., 2012). The rationale behind these influences is that Facebook users who are high in anxious-attachment are likely to maintain a larger number of friends, and to have more intensive relationships, but experience no satisfaction with these relationships. In contrast, Facebook users who are high in avoidant-attachment are expected to have fewer friends, to be less active in maintaining these relationships and to be less emotionally involved with these friends.

Other major personality traits that have been studied in past research include self-esteem, narcissism, conscientiousness, loneliness and self-worth. Users with low levels of self-esteem and high levels of narcissism tend to spend more time on Facebook and post self-promotional Photoshop-enhanced images (Mehdizadeh, 2010). Self-esteem was also found to be negatively correlated with emotional connection with Facebook (Seto, 2012). On average, heavy Facebook users tend to be less conscientious and socially lonely (Ryan and Xenos, 2011). With regards to self-worth it was found that its sources (appearance, outdoing, and others) explain online sharing of photos (Stefanone et al., 2011). Hence, Facebook users low on self-esteem will be more likely than others to self-enhance their image on Facebook, and present higher degrees of false Facebook-self.

Lastly, authenticity is another personality trait which has received attention, because it is seen by many psychology perspectives as an important aspect of well-being (Horney, 1951; Rogers, 1961; Winnicott, 1965; Yalom, 1980; May, 1981) and departures from it are often seen as increasing the likelihood of psychopathologies. Authenticity is the degree to which one is true to his or her own personality, spirit, or character, despite external pressures. Psychodynamic theoreticians, such as Horney (1951) and Winnicott (1965) focused on the internalization of external aspects of life during childhood as something that can lead to self-alienation, while existential theoreticians, such as Yalom (1980) and May (1981) focused particularly on self-alienation as the core of authenticity. Both points of view agree that authenticity is a key influencer of well-being, and that major deviation from it can lead to problems. In the current research we hypothesized that one’s authenticity in the “real world” (outside of Facebook) will mirror into the Facebook arena, and will lead to lower degrees of falseness in one’s Facebook representation.

Ultimately, the growth in Facebook usage seems to result in part from the various benefits it provides to its users; and that these benefits can vary based on individual differences in terms of self-esteem and anxious attachment styles. However, as we argue here, this growth can also fuel the development of false Facebook self-identities. Based on the abovementioned logic, we argue that the development of false Facebook-self is driven, in part by low authenticity and low self-esteem, and that these individual differences can be influenced by one’s upbringing and the negative attachment styles – avoidant and anxious, he or she has developed. We expect that avoidant and anxious styles will reduce one’s self esteem and his or her authenticity because less-authentic behaviors and reduced self-esteem may result from less secured attachment styles as a means to protect one from engaging in relationships in which he or she is perceived to be inferior to others.

#### Research model

**FIGURE 1:**
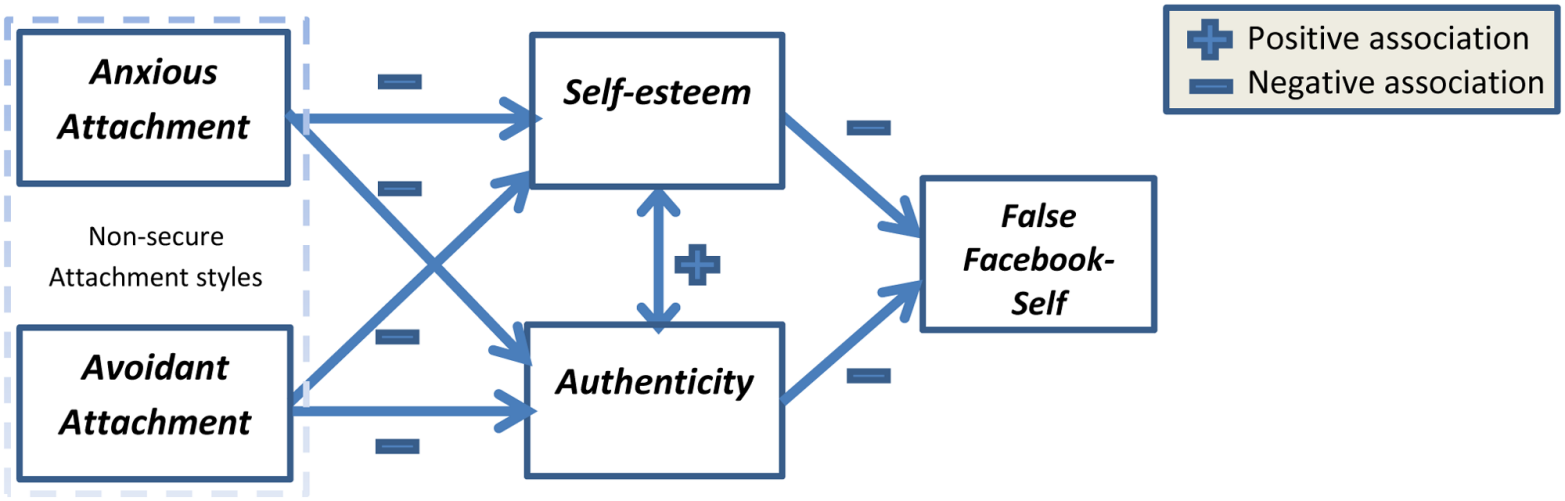
**Research model**.

Given the possible prevalence and importance of the false Facebook-self and its possible adverse consequences, the aim of this study is to analyze key psychological processes leading to a false Facebook-self, with an emphasis on key predictors mentioned in the previous sections. Integrating the possible influences of these predictors on false Facebook-self, we propose the model depicted in **Figure 1**. The model starts with early childhood, where infant’s relationships with his or her main caregiver influence his or her attachment style. As predicted by Bowlby’s (1969) attachment theory, non-secure attachment styles (anxious and avoidant) influence long-term relationships with others, and more importantly, personality characters which relate to self-worth perception and image. In our model we predict that both avoidant and anxious attachment styles negatively influence one’s self-esteem (Arbona and Power, 2003) and authenticity (Gillath et al., 2010) in real life (outside of Facebook). These two personality characteristics, in turn, will negatively influence the creation of a false Facebook-self. That is, people high in non-secure attachment styles are predicted to develop lower general life authenticity and lower general self-esteem. When self-esteem and authenticity are low, we predict that individuals’ self-representation on Facebook will deviate from their true-self; i.e., they will present stronger false Facebook-selves.

## MATERIALS AND METHODS

The study involved 258 participants, 183 females, 62 males, and 13 participants who did not report their gender. All participants were Israeli adults (between the ages of 20 and 65 years old with an average age of 27.04 years and SD = 6.12) with an active account on Facebook. Participants were approached using an online questionnaire (Qualtrics) which was distributed to freshman undergraduate psychology students (*N* = 135), as well as to freshman graduate business students (*N* = 123).The scales used in this study can be found in appendix A. A description of the scales and their reliability scores in the current study are provided below:

(1) Attachment style questionnaire [a short version of the Experiences in Close Relationships Scale (ECR); Brennan et al., 1998] was used to assess adult attachment patterns. The scale included 16 statements such as: “I prefer not to show others how I feel deep down,” “I worry about people leaving me,” “I am comfortable being close to other people.” The items were rated on a 7-point Likert scale from 1 – “Strongly disagree” to 7 – “Strongly agree.” Respondents were asked to rate how they generally experience close relationships. The Anxiety subscale consisted of eight items assessing fear of abandonment, preoccupation with one’s close partner, and fear of rejection. The Avoidance subscale was also composed of eight items, and assessed avoidance of intimacy, discomfort with closeness, and self-reliance.

Based on the classification by Bartholomew and Horowitz (1991), four categories or styles of adult attachment were identified, based on both attachment style levels and median splits – secure (low levels of both avoidance and anxiety attachment), dismissive (high level of avoidance attachment and low level of anxiety attachment), preoccupied (low level of avoidance attachment and high level of anxiety attachment) and fearful (high levels of both avoidance and anxiety attachment). Previous applications of the scale reported coefficient alphas of 0.76–0.84 in the Anxiety subscale and 0.68–0.76 in the Avoidance subscale (Gur, 2006). In the present study, coefficient alphas were 0.80 and 0.75 for the Anxiety and Avoidance subscales, respectively.

(2) Self-esteem questionnaire [Rosenberg Self-Esteem Scale (RSE); Rosenberg, 1965] captured general levels of self-esteem. The scale included 10 statements such as: “I hold a positive attitude toward myself,” “I’m inclined to feel that I’m a failure,” “I feel that I have a number of good qualities.” The items were rated on a 5-point Likert scale from 1 – “Strongly disagree” to 5 – “Strongly agree.” Previous studies have reported coefficient alphas for the RSE scale ranging from 0.72 to 0.88 (Gray-Little et al., 1997). In the present study, coefficient alpha was 0.87.
(3) Authenticity questionnaire (Authenticity Scale; Wood et al., 2008) was used for measuring the authenticity trait of the Facebook users in their real life. The authenticity scale comprised of three sub-scales: authentic living, accepting external Influence and self-alienation. The scale included 12 statements such as: “I think it is better to be yourself than to be popular,” “I don’t know how I really feel inside,” “I am strongly influenced by the opinions of others.” Each of the three sub-scales includes four statements. The items were rated on a 7-point Likert scale from 1 – “does not describe me at all” to 7 – “describes me very well.” The authors reported coefficient alpha of 0.70–0.82 for the authentic Living sub-scale, 0.77–0.84 for the accepting External Influence sub-scale and 0.82–0.84 for the self-Alienation sub-scale. In the present study, coefficient alphas were 0.75, 0.79, and 0.64 correspondingly. The Authenticity scale overall, had a coefficient alpha of 0.65, which is somewhat low but acceptable.
(4) False Facebook-self questionnaire [Perception of “False Self” Scale (POFS); Weir and Jose, 2010] was adapted for this research in order to measure the difference between the perceived day-to-day self and the perceived self within Facebook, as well as the falseness of one’s self on Facebook (regardless of who the user is in real life). The bigger the difference was, the more false one’s Facebook-self was, and to stronger his or deviation from reality was (i.e., strong agreement indicated higher degrees of falseness). Very high false Facebook-self scores can represent a fictional self which may meet DSM criteria for delusional disorder, medium scores of false Facebook-self represent moderately self-exaggerated and enhanced self, and low scores represent a relatively consistent, accurate and reliable depiction of the self on Facebook. Please note that this study treated the false Facebook self as a continuous concept (e.g., like the treatment of addiction scores in past research; see Turel et al., 2014), but future research can find the infliction point after which fictional self is developed, or where clinical treatment is warranted.
(5) The scale included 21 statements such as: “I say what I think on Facebook even if it is different from the opinions of others,” “I cannot express my opinions to others on Facebook,” “I act one way, but want to act a different way on Facebook.” The scale tapped into multiple facets of the false-self: the gap between the true and Facebook self, the gap between the Facebook self and real-life self, and gaps between the signals provided on one’s Facebook page and social expectations. The items were rated on a 5-point Likert scale from 1 – “Strongly disagree” to 5 – “Strongly agree.” The authors reported coefficient alpha of 0.84. In the present study, coefficient alpha was 0.86. The Facebook-self levels measured in this study ranged between 1.14 and 3.90 on a 5-point scale, with an average of 2.43 and a standard deviation of 0.50. Higher levels of false Facebook-self indicated large gaps between the true self and the one presented on Facebook.
(6) Demographic questionnaire captured sex, age, marital status, education levels and income levels.

### ANALYSIS AND RESULTS

Several analyses were performed in order to test the proposed associations. First, the role of demographics was examined, and largely ruled out. Next, a descriptive assessment of the variables was performed, and demonstrated a preliminary viability of the proposed associations. Next, a sequence of ANOVAs was used for establishing potential associations between the pertinent individual differences and the Facebook-self. Lastly, a structural equation model depicting all hypothesized effects was estimated.

#### Sample composition – demographic variables

The sample included 258 participants, out of whom 183 were women (74.7%) and 62 men (25.3%). Thirteen participants did not disclose their sex. The vast majority of the participants (74.1%) were single followed by 22.3% who were married. The majority of the sample (52.3%) held either a Bachelor’s or a Master’s degree.

To test the contribution of the demographic characteristics to the research’s main variables, one-way ANOVA tests were conducted. Levels of anxious attachment [*F*(1,243) = 9.713, *p* ≤ 0.05] and self-esteem [*F*(1,243) = 7.933, *p* ≤ 0.05] were found to be different for the different sexes, where women had higher anxious attachment levels (*M* = 3.26, SD = 1.04) compared to men (*M* = 2.79, SD=1.0), as well as lower self-esteem (*M* = 3.95, SD = 0.64) compared to men (*M* = 4.20, SD = 0.49). Levels of anxious attachment [*F*(2,245) = 3.782, *p* ≤ 0.05] were found to be different also for the different marital status categories. To further examine the difference between the categories, a *post hoc* Scheffe test was conducted with significance level of *p* < 0.05. The results showed that there is a significant difference in anxious attachment between single participants (*M* = 3.23, SD = 1.08) and married ones (*M* = 2.80, SD = 0.88), and that single participants had higher levels of anxious attachment.

Levels of anxious attachment [*F*(2,245) = 4.85, *p* ≤ 0.05] were found to be different also for the different education levels. To further examine the difference between the categories, a *post hoc* Scheffe test was conducted with significance level of *p* < 0.05. The results showed that there is a significant difference in anxious attachment between participants with high school education (*M* = 3.35, SD = 1.0) and participants with a bachelor degree (*M* = 2.94, SD = 1.12). Levels of anxious attachment [*F*(5,243) = 3.72, *p* ≤ 0.05] and self-esteem [*F*(5,243) = 4.27, *p* ≤ 0.05] were found to be different for the different salary levels. To further examine the difference between the categories, a *post hoc* Scheffe test was conducted with significance level of *p* < 0.05. The results showed that there is a significant difference in anxious attachment between the lowest salary band participants (*M* = 3.79, SD = 1.13) and the third salary band (*M* = 2.93, SD = 0.98). There is also a significant difference in self-esteem between the lowest salary band participants (*M* = 3.60, SD = 0.80), the third salary band (*M* = 4.14, SD = 0.50) and the fourth salary band (*M* = 4.11, SD = 0.47).

Other than these reported differences, there was no additional contribution of the demographic characteristics to the model’s variables, and most importantly to the reported levels of false Facebook-self. Hence, they were not included as predictors in the next analysis.

#### Predictors of the false Facebook-self

**Table 1** displays the means, SDs, correlations between the variables of the model and their significance, as well as the reliability scores (Cronbach Alphas) for the scales.

**Table 1:** Means, SD, Cronbach alpha and correlations between the variables^†^.

*n* = 252	Mean (SD)	1	2	3	4	5
(1) Attachment-anxious	3.14 (1.04)	*0.8*				
(2) Attachment-avoidant	3.01 (0.93)	0.064	*0.75*			
(3) Self esteem	4.01 (0.61)	-0.562*	-0.240*	*0.87*		
(4) Authenticity	5.56 (0.77)	-0.438*	-0.214*	0.614*	*0.65*	
(5) Facebook-self	2.32 (0.50)	0.312*	0.250*	-0.428*	-0.494*	*0.86*

*Correlation is significant at the 0.01 level (two-tailed). ^†^Cronbach alphas are on the diagonal (italicized).

***Personality characteristics’ contribution to a false Facebook-self.*** In order to understand the indirect contribution of attachment styles to the false Facebook-self based on the classification by Bartholomew and Horowitz (1991), two dummy variables were created using a median split: anxious and avoidant, dividing the anxious attachment style and avoidant attachment style variables into two categories (low and high) using the median as the cutoff point.

In order to establish possible association between Facebook-self scores and the pertinent attachment styles, Facebook-self scores were subjected to a two-way ANOVA having two levels of anxious attachment (low, high) and two levels of avoidant attachment (low, high). All effects were statistically significant at the 0.05 significance level. The main effect of anxious attachment yielded *F*(1,248) = 11.01, *p* < 0.001, indicating that the mean false Facebook-self score was significantly greater for high anxious-attachment participants (*M* = 2.42, SD = 0.47) than for low anxious-attachment participants (*M* = 2.22, SD = 0.52). The main effect of avoidant attachment yielded *F*(1,248) = 12.11, *p* < 0.001, indicating that the mean Facebook-self score was significantly greater for high avoidant-attachment participants (*M* = 2.44, SD = 0.05) than for low avoidant-attachment participants (*M* = 2.44, SD = 0.05). The interaction effect was non-significant [*F*(1,248) = 0.833, *p* > 0.05], and participants with both high levels of anxious-attachment and high levels of avoidant-attachment had a mean Facebook-self score of 2.51 (SD = 0.42).

**FIGURE 2:**
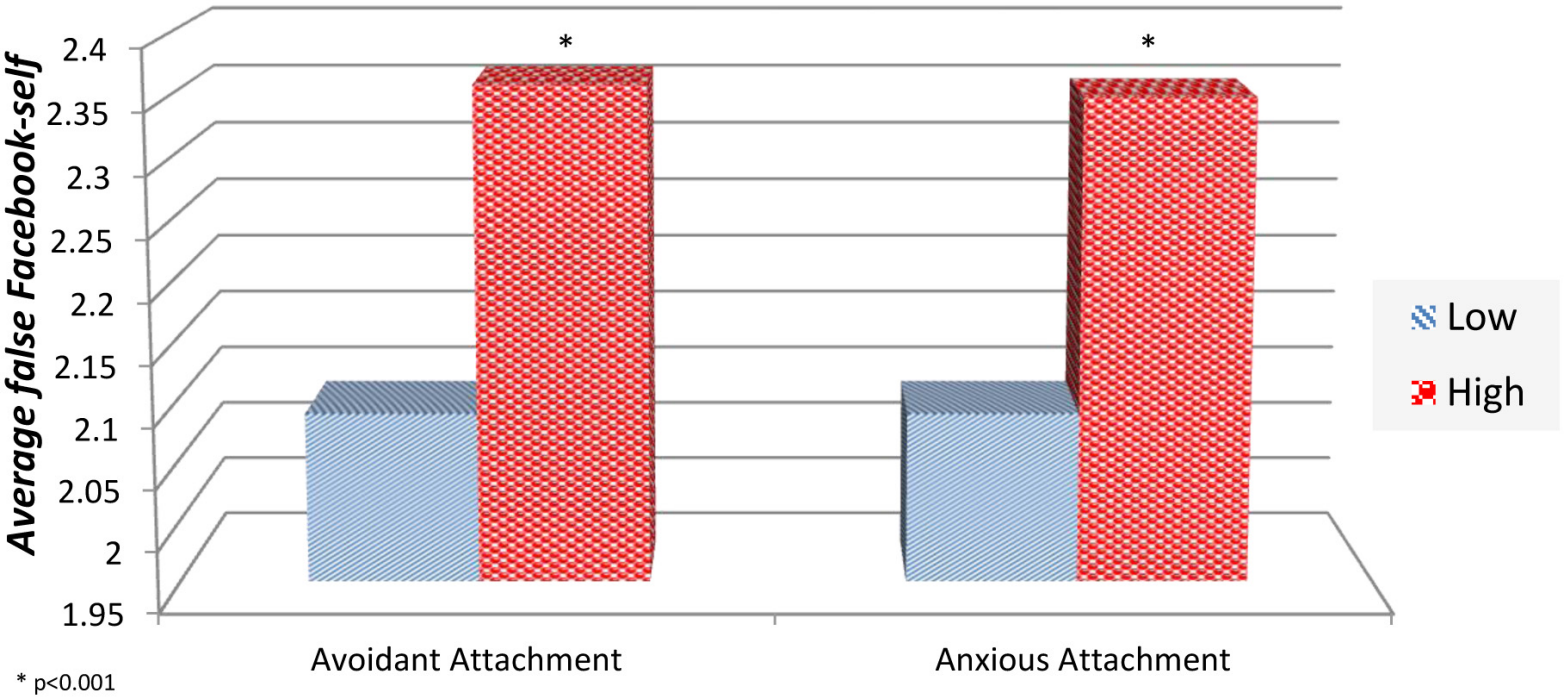
**Average false Facebook-self levels in each avoidant and anxious attachment style categories**.

**Figure 2** shows the average Facebook-self levels in each of the avoidant and anxious attachment style categories.

After preliminarily testing the indirect total contribution of attachment style to the appearance of a false Facebook-self, we wanted to preliminarily examine the contribution of the individual-difference variables which we hypothesize to directly influence false Facebook-self levels (Self-esteem and authenticity). Toward this end, two dummy variables were created for each personality characteristic: low and high, using the median as the cutoff point (i.e., median split).

**FIGURE 3:**
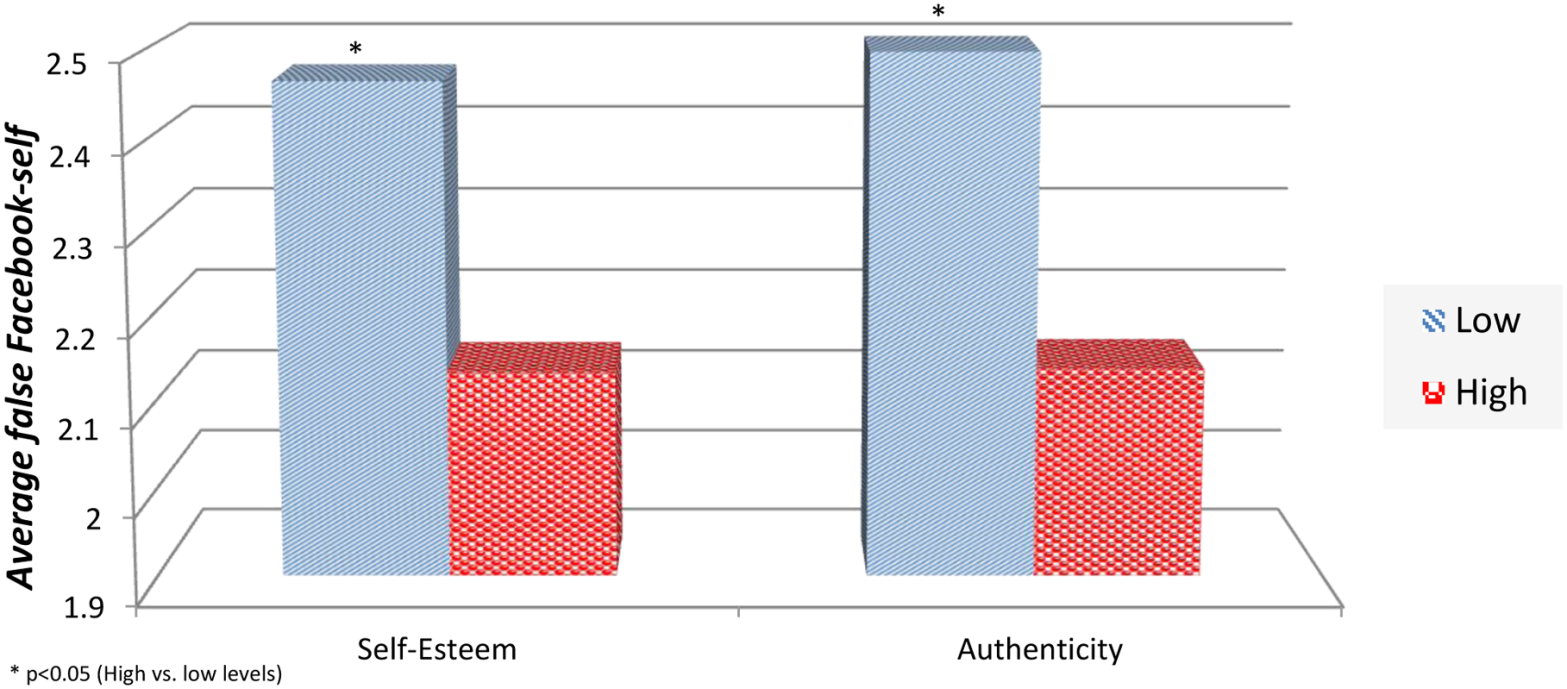
**Average false Facebook-self levels in each self-esteem and authenticity categories**.

Next, two separate one-way ANOVA tests were conducted. These tests showed that the both effects of self-esteem [*F*(1,250) = 29.617, *p* ≤ 0.05] and authenticity [*F* (1,250) = 36.324, *p* ≤ 0.05] were statistically significant. **Figure 3** shows the average Facebook-self levels in each self-esteem and authenticity category. Based on these results, the higher levels of false Facebook-self appear in individuals with lower levels of self-esteem and authenticity.

**FIGURE 4:**
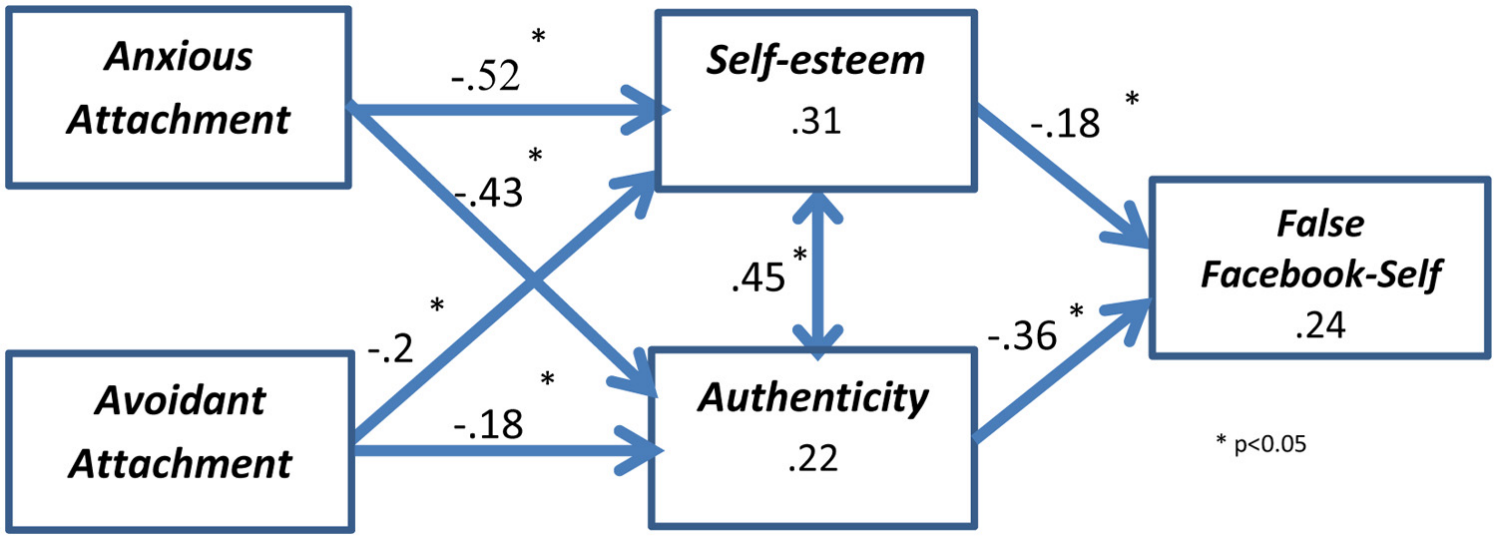
**Structural model**.

Following these preliminary analyses, we sought to examine the suggested research model as a whole, which explicates also links among individual characteristic’s and accounts for shared variance among such characteristics as well as for the hypothesized non-direct effects. We did that in order to examine the two-stage model we theorized– the influence of early-child attachment styles on personality characteristics (self-esteem and authenticity) as the first stage, and the influence of these personality characteristics on the creation of a false Facebook-self, as the second stage. This chronological process representation captures the intricacies of interactions among our predictors and the ultimate outcome variable – false Facebook-self. To this end, we used path modeling with maximum-likelihood estimations in the structural equation modeling (SEM) facilities of AMOS 20.0. The hypothesized model indicated good fit to the data (x_[3]_^2^ = 7.902, *p* > 0.05, x^2^ /df = 2.63, NFI = 0.97, CFI = 0.98, RMSEA = 0.08). The standardized path coefficients, their levels of significance (above the arrows) and the squared multiple correlations (SMCs, in the boxes) are provided in **Figure 4**. As can be seen, all hypothesized paths were significant and in the expected direction. Hence, our theory as expressed in the model was supported. Both attachment styles had negative effects on self-esteem and authenticity, which in turn had negative effects on the false Facebook-self.

## DISCUSSION

The overall aims of this study were to introduce the concept of false Facebook self as a potential driver of psychological problems and to examine key psychological factors driving the creation and maintenance of this false Facebook-self. Based on our findings, some degree of false Facebook-self was reported by the research participants (*M* = 2.32 on a 5-point Likert scale, SD = 0.5), which means that on average, users believe that their Facebook-self differs from who they really are and represents in part a person which adheres to social pressures, exaggerates positive facets, and downplays weaknesses. Although the average score is below the middle point (three on a 5-point Likert scale), there were 19 participants (7.5% of valid responses) who reported higher false-self than the middle point. These levels seem reasonable because the participants in this research were relatively functional students, and consequently did not present extreme levels of false Facebook-self, which may be even perceived as delusional and require treatment. Future research can attempt to find such individuals and explore their clinical needs. In addition, it is possible that our sample included more cases of severe false Facebook self, but the Facebook self was underreported due to social desirability bias. This is a point which merits further research.

The Facebook-self represents the way a Facebook user is presenting himself or herself to the Facebook community. We focused on the falseness of one’s Facebook-self as an indicator that can shed light on his or her Facebook behavior and possible psychological problems. Many studies have analyzed the characteristics and behaviors of Facebook users, but to the best of our knowledge there is no research analyzing the falseness of the Facebook users, and more specifically the personality factors that contribute to this falseness.

In this study, individual differences related to self-esteem and authenticity in real-life were found to reduce the magnitude of one’s false Facebook-self. Both avoidant and anxious attachment styles were found to negatively impact these individual differences, and by doing so, indirectly influence the formation of false Facebook-self. Hence, our results show that the false Facebook-self is driven, in part, by shortcomings in one’s personality, which may relate to his or her upbringing and the consequent social traits he or she has developed. Specifically, false Facebook-self is enhanced when self-esteem and general authenticity are low, and these traits tend to be lower among individuals with avoidant and anxious attachment styles. This is consistent with Bowlby’s (1969) attachment theory and extends it to an online context. It also extends findings regarding the connection between insecure attachment styles and dishonest behaviors, such as lying and cheating (Gillath et al., 2010), as well as connections between insecure attachment and authenticity in real life (Marshall and Smith, 1986), to an online context. We hence in this study extend our knowledge regarding oﬄine behaviors to the growing in prevalence online world of social interactions.

This study’s findings also point to the fact that Facebook and the range of positive social reward it provides to users has created a playground for people that have difficulties in interacting with others, in which they can rationally chose to present a false self-image. These users who may have had difficulties in their early years of development tend to create a non-secure attachment style, have lower self-esteem and are often not very authentic in their real life. These “disadvantages” do not disappear in the virtual world, but the perceived distance from others in the virtual world gives these users the ability to communicate with others; an ability which they usually lack in face-to-face environments. Still, in many cases these social interactions on social networking sites are done using false self-representations which can be relatively far from their real selves. As argued by false-self theories, this divergence may be a source for various psychological problems.

It is important to note that false-self presentations is not necessarily a concern for all users, as evident in the variation of false Facebook-self scores we obtained in this study, and the many users with low self-reported false Facebook-self scores. Some users may have the exact same profile as the “false” ones, but present a true Facebook-self and can leverage the positive benefits of the online social networking site to a fuller extent. In contrast, others may have more extreme cases of false Facebook-self, and these individuals may require treatment due to possible adverse consequences of this falseness, as prescribed by false-self theories. The current study examined a convenience sample of functional young-adults who perhaps not surprisingly presented low-medium levels of false Facebook-self. These individuals, excluding some extreme cases, may not require treatment. However, further research is needed for substantiating this conclusion, and perhaps future research can (1) detect more severe cases, (2) perhaps examine whether treatment is needed, and(2) test the efficacy of various treatments with regards to dealing with one’s level of false Facebook self.

## CONCLUSION

The unique contribution of this study is in introducing and emphasizing the importance of the false Facebook-self and the factors that contribute to its appearance. We suggest that false Facebook-self is an important phenomenon to study because it can serve as a gateway issue to more problematic behaviors which may lead to psychological problems and even pathologies, such as Facebook addiction. While this proposition requires further research, the results of this study show the psychological characteristics (attachment, authenticity, and self-esteem) that can lead to the creation of a false Facebook-self. By doing so, we pave the way for further research on this topic.

These findings have possible implication for both diagnosis and treatment of problematic Facebook use. The findings can also be used for prevention and psycho-education purposes. Such psycho-education efforts should reduce the negative circumstances leading to a false presentation within Facebook. In addition Facebook users who will be found to have high levels of false Facebook-self might have the opportunity to go into prevention treatment, where the risks of mal-usage of Facebook will be explored with them. These suggested solutions should be explored in future research, since the current research design does not cater to examining prevention treatments and their effects.

Additional possible treatment procedures should be examined in future research. For example, future research can examine the efficacy of cognitive beliefs challenging of cognitions which lead to the presentation of the false Facebook-self, or the efficacy of “replacement” of unhealthy portions of the false Facebook-self with more functional portions, and others. In addition to the cognitive treatment, other treatments can be examined in future research, especially when extreme cases of false Facebook self are detected. Based on theoretical similarities between the narcissistic personality structure and the characteristics of false Facebook-self found in this study, techniques that may work and require further research include behavioral treatment and psychodynamic therapy. We hence call for future research to examine the need for and efficacy of such techniques.

Ultimately, we believe that future studies should further explore the concept of false Facebook self, its antecedent, consequences and treatments (Tarafdar et al., 2013; D’Arcy et al., 2014). Such studies are essential because the use of social networking sites has become the main rather than secondary means of social interaction for many people. At the same time, problematic and excessive use of such sites can be associated with pathologies (Turel et al., 2014), and prevention and teratement techniques may be in order (Turel et al., 2015). This study has made initial strides in this direction, by focusing on the Facebook-self concept and its antecedents. We hence call for more research on this important, yet underexplored topic.

## Conflict of Interest Statement

The authors declare that the research was conducted in the absence of any commercial or financial relationships that could be construed as a potential conflict of interest.
