# Corrigendum: The “Facebook-self”: characteristics and psychological predictors of false self-presentation on Facebook

**DOI:** 10.3389/fpsyg.2018.00652

**Published:** 2018-04-30

**Authors:** Oren Gil-Or, Yossi Levi-Belz, Ofir Turel

**Affiliations:** ^1^College of Management Academic Studies, Rishon LeZion, Israel; ^2^The Academic College of Tel Aviv Yafo, Tel Aviv Yafo, Israel; ^3^Department of Behavioral Sciences, Ruppin Academic Center, Emek Hefer, Israel; ^4^Information Systems and Decision Sciences, Mihaylo College of Business and Economics, California State University, Fullerton, CA, United States; ^5^Psychology, Brain and Creativity Institute, University of Southern California, Los Angeles, CA, United States

**Keywords:** social networking sites, Facebook, false self, attachment theory, self-esteem, authenticity

In the original article, there was an error. The original text on page 6 is: “The main effect of avoidant attachment yielded *F*_(1, 248)_ = 12.11, *p* < 0.001, indicating that the mean Facebook-self score was significantly greater for high avoidant-attachment participants (*M* = 2.44, *SD* = 0.05) than for low avoidant-attachment participants (*M* = 2.44, *SD* = 0.05).”

The corrected text on page 6, Analysis and Results section, Sub-section of “Predictors of the false Facebook-self is: “The main effect of avoidant attachment yielded *F*_(1, 248)_ = 12.11, *p* < 0.001, indicating that the mean Facebook-self score was significantly greater for high avoidant-attachment participants (*M* = 2.43, *SD* = 0.44) than for low avoidant-attachment participants (*M* = 2.22, *SD* = 0.53).”

The authors apologize for this error and state that this does not change the scientific conclusions of the article in any way.

The original article has been updated.

## Conflict of interest statement

The authors declare that the research was conducted in the absence of any commercial or financial relationships that could be construed as a potential conflict of interest.

