# Supplementary material for: The “Facebook-self”: characteristics and psychological predictors of false self-presentation on Facebook
Source: Front Psychol. 2015 Feb 17;6:99. doi: 10.3389/fpsyg.2015.00099 (PMC4330900; doi:10.3389/fpsyg.2015.00099)
Supplement: Supplementary file 1 [file Data_Sheet_1.DOCX]

**Appendix A – Questionnaire items**

***Attachment Style***

1. I prefer not to show others how I feel deep down.
2. I worry about being abandoned.
3. I am very comfortable being close to other people.
4. I’m getting worried when thinking about my relationships with other people.
5. When other people start to get close to me I find myself pulling away.
6. I worry a fair amount about losing close people.
7. I get uncomfortable when a other people wants to be too close to me.
8. I worry about being alone.
9. I feel comfortable sharing my private thoughts and feelings with other people.
10. My desire to be very close sometimes scares people away.
11. I tell people that are close to me just about everything.
12. I find that other people don’t want to get as close to me as I would like to get close to them.
13. I usually discuss my problems and concerns with people that are close to me.
14. I get frustrated when other people are not around as much as I would like.
15. I get frustrated if other people are not available when I need them.
16. It helps to turn to other people in times of need.

(*) Items 3, 9, 11, 13 and 16 will need to be reverse-keyed before you compute the average.

***Self-Esteem***

1. On the whole, I am satisfied with myself.
2. At times I think I am no good at all.
3. I feel that I have a number of good qualities.
4. I am able to do things as well as most other people.
5. I feel I do not have much to be proud of.
6. I certainly feel useless at times.
7. I feel that I am a person of worth, at least on an equal plane with others.
8. I wish I could have more respect for myself.
9. All in all, I am inclined to feel that I am a failure.
10. I take a positive attitude toward myself.

(*) Items 2,5,6,8 and 9 will need to be reverse-keyed before you compute the average.

***Authenticity***

1. I think it is better to be yourself, than to be popular.
2. I don’t know how I really feel inside.
3. I am strongly influenced by the opinions of others.
4. I usually do what other people tell me to do.
5. I always feel I need to do what others expect me to do.
6. Other people influence me greatly.
7. I feel as if I don’t know myself very well.
8. I always stand by what I believe in.
9. I am true to myself in most situations.
10. I feel out of touch with the ‘real me.’
11. I live in accordance with my values and beliefs.
12. I feel alienated from myself.

***False Facebook-Self***

1. There is a gap between my self-presentation on Facebook and my day-to-day self-presentation
2. People that know me on Facebook will be surprised how I am in real life
3. My activities and responses on Facebook are similar to those in real life
4. Sometimes I figure out I respond differently to people on Facebook than in real life
5. A large number of my Facebook friends are not my friends in real life
6. I say what I think in Facebook even if it is different from the opinions of others
7. I cannot express my opinions to others on Facebook
8. I act one way on Facebook, but want to act a different way
9. I don’t let people see the real me on Facebook
10. My thoughts are not important to others on Facebook
11. I hide the real me on Facebook by looking like others
12. I act on Facebook in ways that express who I really am
13. I hide my true feelings on Facebook if I think they will upset others
14. I tend to say one thing on Facebook even when I think another
15. I can talk openly to others on Facebook about my feelings
16. I stay quiet on Facebook when I don’t agree with other people
17. I don’t like to look different from other people on Facebook
18. If people on Facebook knew what I was really like on the inside, they would not like me
19. Other Facebook users’ feelings are more important than mine
20. I spend a lot of time thinking about how other people on Facebook see me
21. What I openly say on Facebook is different from what I think deep inside

(*) Items 3,6,12 and 15 will need to be reverse-keyed before you compute the average.
